# Atlanta Society of Medicine

**Published:** 1897-08

**Authors:** T. H. Hancock

**Affiliations:** Secretary; Atlanta, Ga.


### *SOCIETY REPORTS.*

### ATLANTA SOCIETY OF MEDICINE.

Atlanta, Ga., July 6, 1897.

The meeting was called to order by the President, Dr. G. H. Noble.

The following members were present: Drs. Willis Westmoreland, Noble, Duncan, Goldsmith, Smith, Divine, McRae, Hancock.

The minutes of the previous meeting were read and approved.

Dr. Willis Westmoreland read a paper on "Carcinoma of the Breast." After reading the paper Dr. Westmoreland made the following remarks:

In performing the operation it is a difficult matter to be sure of removing all the glands. I have here the picture of a boy, a tubercular case, which illustrates how high in the triangle of the sternum and clavicle we may have glands, and we should be careful to go high enough in our operations to remove them.

In regard to the negro, the statement of Dr. Warren, in his recent *Surgical Pathology*, that carcinoma does not occur in the negro, is not true. I do not think it is as frequent, but in the last two years and a half we have had at least half a dozen cases.

I am very much in favor of the Halsted operation, but it is one which a man does not want to attempt unless he has had some surgical experience, or has practiced on a cadaver. I see that Dr. McBurney has made the suggestion that instead of beginning the incision from the top of the shoulder, to begin at the lower portion of the axilla. He claims that it can be done just as well, and in addition to that, if the woman should desire, she can afterward wear a low dress and not show the scar.

In closing up the wound my experience has been that it is best not to use any traction, but simply hold the skin in position. I think you get better results than when you attempt to bring the flaps down along the line of incision.

My experience has been that it is better to remove the pectoral, major and minor muscles entire, for the patient does not afterward complain of the stiffness which you otherwise have, and also you can preserve the nerves better in operating.

In regard to the skin-grafting, that is, the time for the wet dressing, they should not be removed too soon. It has been pointed out by Schultz that if they are taken off too early you may have a case in connection with the surface from which the grafts are taken. Schultz says that the wet dressing should be removed only after from ten to fourteen days. One case on which we operated returned to-day, and it presented the keloid appearance. The wet dressing on this was removed after about the first week.

*Dr. McRae:* The subject of carcinoma is one of very great and increasing interest. I do not think anybody has done more in recent years to develop the operation than Dr. Halsted, and his unprecedented results show what may be done in this class of cases. I do not think there is any question that, under the most favorable conditions, it is the operation of choice. However, the Halsted and other similar operations can be done only by experienced operators. I do not think it is the operation for unfavorable surroundings or unfavorable conditions. The operation is very formidable; there are a large number of vessels which bleed freely, and it is an operation which needs a great deal of help. For these reasons it must be done by operators of experience. I think this is a point that is to be emphasized, as the favorable reports are apt to lead young men into attempting it. I have done this operation a few times, but do it with dread every time. The point that Dr. Westmoreland referred to, of clearing out the axilla when the muscles have been removed, is one that every one can appreciate. Another point that is of importance is the clamping and section of a great many of the axillary vessels after they have been ligated. You may lose practically no blood from these operations by this method. I have recently done this operation under very unfavorable conditions, in a country house, with men who had never seen the operation done. The patient was a very large woman and the operation made an enormous hole. However, she got well, although at the time I had serious doubts.

*Dr. Goldsmith:* Dr. Westmoreland, in mentioning his statistics, has brought up some valuable points. However, he overlooked one in regard to the mortality in connection with skin-grafting. Years ago the complete operation was not attempted on account of the large surface left exposed, but of late years Hurst's method of skin-grafting has overcome this objection, and in this way I think has reduced the mortality. The operations mentioned by Dr. Westmoreland were clinical cases, and were done at the college, and were not entirely under our control. The patients were carried home after the operations.

*Dr. Noble:* I have been very much interested along this line of work, and fully indorse everything that has been brought forward. However, there is one point I would mention, and that is the time at which the radical operation should be done. Being formidable, the general practitioner is rather slow to refer the patient to the surgeon for radical operation. The tendency is to apply it only to the advanced cases, but these are the cases which give the most unfavorable results. If the profession at large could be made to understand that the radical operation should not be delayed, then the recurrence would be much rarer. As far as I know, I was the first to do the operation in this part of the country. However, the second time I made the incision from the breast to the clavicle and then down into the axilla, and this way I found to be much easier and subject to less loss of blood.

*Dr. Westmoreland:* In speaking of the late time in which the patient is sent to you, I had two cases last winter which illustrated this. One woman had shown carcinoma for four or five months before she was finally sent to me by her physician. The other woman was pregnant and she was allowed to go through this before the operation was performed, and of course this simply meant her death-warrant, as she died within three months.

I was agreeably surprised with my first Halsted operation; although it was formidable, yet I had very little trouble. I was fortunate in having a slender woman to operate upon. The plan I followed was, beginning at the sternum, to carry the first incision obliquely up to the clavicle, and then go along the clavicle and expose the vein and use this as my guide in going into the axilla. In this way you are sure to care for the blood-vessels. In this connection I would say that I am very careful to have sharp knives and know exactly how sharp they are, so that I can tell how much force to exert. I litigate at once after clasping with the forceps, for if you have a large number in the wound at one time you may have trouble by the forceps being jerked off.

In regard to the shock, I use very hot towels to cover the exposed surface, and this also prevents oozing. My experience has been that I lose less blood in these operations than I do in the other operations. While the pulse goes down, yet it comes up very soon because there is no loss of blood.

In all these cases, as a preliminary precaution, I put the patient on strychnine, if the patient comes to me soon enough before the operation, and immediately preceding the operation I give them an injection of one-thirtieth of a grain. I have an assistant to watch the pulse and give strychnine during the operation, if necessary. The way to get the best results is to operate early, as Dr. Noble says, and we should educate the general practitioners and have them see that the cases which come under their observation are operated upon early; most of these general practitioners believe as the old surgeons did, that the operation is simply temporary relief.

One thing I notice in Halsted's reports is the number of cases which he dismisses from the hospital with the statement that the wound has nicely granulated. I am of the opinion that granulation tissue predisposes to a recurrence. In these cases I found also that within two weeks the skin begins to adhere to the chest around the border of the wound and here contract, as a result of the slight granulation, and I think it is best to scrape off these granulations before putting on the skin grafts. I think this is better than the method of Willy Meyer, who puts them on over the granulations. By scraping off these granulations you remove a very apt source of infection, and an application of hot cloths stops the oozing and gives an excellent surface for the grafts.

I have been trying the skin grafts in cases of keloids, and with success that was surprising to me. I had tried healing by granulation and moist blood-clot, but they were very unsatisfactory, as there would be recurrence. However, with the skin grafts I think we are going to have good results, so far as recurrence is concerned.

### REPORT OF CASES.

*Dr. McRae:* I had a case of appendicitis recently, which was of unusual interest in one of its features. The patient was the daughter of one of the most prominent physicians in Macon, and I operated on her last Sunday a week ago, assisted by Drs. McHatton, Williams, Hall, and Derry. She had been sick about twenty-five days, and was first taken with violent pains in her stomach, accompanied by vomiting. The pain was localized in the beginning and was relieved with morphia, which was given to her regularly. This trouble subsided, but was followed by an enlargement of the parotid glands. She suffered severe pain with those for about two weeks, and after this subsided she had a violent fit of coughing, and immediately there began to develop rapidly an abscess in the region of the appendix. The enlargement of the parotid glands was very peculiar; I believe Osler mentions metastasis in abdominal trouble.

When I operated she had considerable temperature. She had been taking for twenty-five days from one to two grains of morphia. The operation was done the hottest day of this year, and the temperature of the room was 98. I found a large abscess, which I simply cleaned out thoroughly and drained. The patient has had a very tedious time of it since the operation, and for several days it looked as though she would die. One of the submaxillary glands had also enlarged, but I had a letter yesterday stating that this had subsided, and I heard to-day that the temperature was normal and the condition favorable.

Now, I made one mistake in this operation which made the condition unfavorable since the operation. I have such a horror of morphia being continued after an operation that I refused to operate without the withdrawal of the morphia. The complete withdrawal of this resulted in such a nervous and delirious condition that I had to continue it. I think it would have been better to have kept it up without even temporary withdrawal. The operation was followed by exhausting diarrhea as a result of the withdrawal of the morphia.

*Dr. Westmoreland:* In regard to the enlarged parotid, I have seen two cases following typhoid fever, and Welch reports thirty cases following abdominal trouble, and in all these I think he has one or two cases resulting from appendicitis.

T. H. HANCOCK, M.D., Secretary.

